# Distinct Roles of Cdc42 in Thymopoiesis and Effector and Memory T Cell Differentiation

**DOI:** 10.1371/journal.pone.0018002

**Published:** 2011-03-24

**Authors:** Fukun Guo, Shuangmin Zhang, Pulak Tripathi, Jochen Mattner, James Phelan, Alyssa Sproles, Jun Mo, Marsha Wills-Karp, H. Leighton Grimes, David Hildeman, Yi Zheng

**Affiliations:** 1 Division of Experimental Hematology and Cancer Biology, Children's Hospital Research Foundation, Cincinnati, Ohio, United States of America; 2 Division of Immunobiology, Children's Hospital Research Foundation, Cincinnati, Ohio, United States of America; 3 Microbiology Institute-Clinical Microbiology, Immunology and Hygiene, Universitätsklinikum Erlangen und Friedrich-Alexander Universität Erlangen-Nürnberg, Erlangen, Germany; 4 Division of Pathology, Children's Hospital Research Foundation, Cincinnati, Ohio, United States of America; University of Georgia, United States of America

## Abstract

Cdc42 of the Rho GTPase family has been implicated in cell actin organization, proliferation, survival, and migration but its physiological role is likely cell-type specific. By a T cell-specific deletion of Cdc42 in mouse, we have recently shown that Cdc42 maintains naïve T cell homeostasis through promoting cell survival and suppressing T cell activation. Here we have further investigated the involvement of Cdc42 in multiple stages of T cell differentiation. We found that in Cdc42^−/−^ thymus, positive selection of CD4^+^CD8^+^ double-positive thymocytes was defective, CD4^+^ and CD8^+^ single-positive thymocytes were impaired in migration and showed an increase in cell apoptosis triggered by anti-CD3/-CD28 antibodies, and thymocytes were hyporesponsive to anti-CD3/-CD28-induced cell proliferation and hyperresponsive to anti-CD3/-CD28-stimulated MAP kinase activation. At the periphery, Cdc42-deficient naive T cells displayed an impaired actin polymerization and TCR clustering during the formation of mature immunological synapse, and showed an enhanced differentiation to Th1 and CD8^+^ effector and memory cells in vitro and in vivo. Finally, Cdc42^−/−^ mice exhibited exacerbated liver damage in an induced autoimmune disease model. Collectively, these data establish that Cdc42 is critically involved in thymopoiesis and plays a restrictive role in effector and memory T cell differentiation and autoimmunity.

## Introduction

T cell development in thymus proceeds through a series of differentiation stages. The most immature populations in thymus comprise CD4^−^CD8^−^ double-negative (DN) thymocytes. The differentiation of DN thymocytes to CD4^+^CD8^+^ double-positive (DP) cells is dependent on the expression and rearrangement of TCRβ and TCRα. DP cells further undergo positive and negative selection, and differentiate to CD4^+^ or CD8^+^ single-positive (SP) T cells. CD4^+^ or CD8^+^ SP T cells migrate to peripheral tissues, e.g. spleen and peripheral blood, where they are maintained as naïve T cells [1].

Upon recognition of peptide-MHC complex on antigen-presenting cells (APC), naïve T cells undergo actin cytoskeletal rearrangement, TCR clustering, and formation of immunological synapse (IS). These cellular events elicit a cascade of intracellular signaling changes including activation of ZAP70 and LAT and subsequent ERK, JNK and p38 MAP kinases, leading to naïve T cell clonal expansion and differentiation into effector and memory cells [2].

There are several types of CD4^+^ effector cells, among which T helper (Th) 1 and 2 are the best studied [3]. Th1 and Th2 cells exert their immune functions through secretion of distinct patterns of cytokines: Th1 cells mediate clearance of intracellular pathogens by producing IFN-γ and TNF-α while Th2 cells are involved in elimination of parasitic organisms by secreting IL-4, IL-5, and IL-13 [3], [4], [5]. On the other hand, cytotoxic CD8^+^ effector cells play essential roles in the protection against intracellular pathogens and tumor cells by generating IFN-γ, TNF-α, granzymes, perforin, and FAS ligand (FasL) [6]. Aberrant cytokine production is involved in the pathogenesis of a variety of autoimmune diseases. For example, IFN-γ contributes to the development of experimental autoimmune myasthenia gravis and liver damage in a liver-specific autoimmune disease model induced by alphaproteobacterium Novosphingobium aromaticivorans (N. aro) [7], [8], [9]. A small fraction of effector cells can further differentiate into memory cells, which are major players in recall immune responses [10]. CD4^+^ memory cells are generally thought to maintain similar cytokine expression patterns of their predecessors [10].

Cdc42 of the Rho GTPase family is an intracellular signal transducer that cycles between an inactive GDP-bound form and an active GTP-bound form under tight regulation [11]. Mostly by overexpression of dominant active or negative mutants, Cdc42 has been shown to regulate actin cytoskeleton reorganization, cell migration, proliferation, and survival [11]. In T cells, overexpression of a dominant mutant suggests that Cdc42 plays a role in actin and tubulin cytoskeleton polarization, migration, and in development [12], [13], [14], [15]. However, this approach is hampered by its nonspecific nature, as dominant mutants of Cdc42 may affect other Rho GTPases [16]. Indeed, distinct cell functions of Cdc42 have been observed in studies of Cdc42 knockout mouse models. For example, contrary to the prevailing view that Cdc42 promotes cell growth and survival, hematopoietic stem cells (HSCs) and HSC-derived myeloid cells deficient in Cdc42 exhibit hyperproliferative properties, and Cdc42-deficient HSCs do not display survival defects, whereas Cdc42-deficient myeloid cells show enhanced survival [17], [18]. Further, Cdc42-deficient fibroblastoid cells and B lymphocytes do not show migratory defects, whereas primary fibroblasts and neutrophils display a dependence on Cdc42 for cell migration [19], [20], [21], [22]. Thus, defining the physiologic role of Cdc42 requires genetic and cell type-specific studies.

To examine the physiological contribution of Cdc42 in T cells, we have generated a Lck-cre driven T cell-specific Cdc42 conditional knockout mouse model. By characterizing this model, we have recently reported that Cdc42 is required for coordinating IL-7R-mediated T cell survival and TCR-mediated T cell activation in maintaining naïve T cell homeostasis [11]. To define the role of Cdc42 in multiple stages of T cell development, in the present study we have examined thymopoiesis, IS formation in peripheral naïve T cell activation, and subsequent effector and memory T cell differentiation, in Cdc42^−/−^ mice. We demonstrate that Cdc42 is required for thymocyte development by impact on positive selection, cell migration, and proliferation. While essential for TCR clustering and actin polarization in the course of mature IS formation, Cdc42 suppresses CD4^+^ Th1 (but not Th2) and CD8^+^ effector and memory T cell differentiation and autoimmunity. These systematic studies suggest a stage-specific role of Cdc42 in T cell developmental cascade.

## Results

### Cdc42 deficiency causes defective positive selection in thymocytes

**Figure 1:**
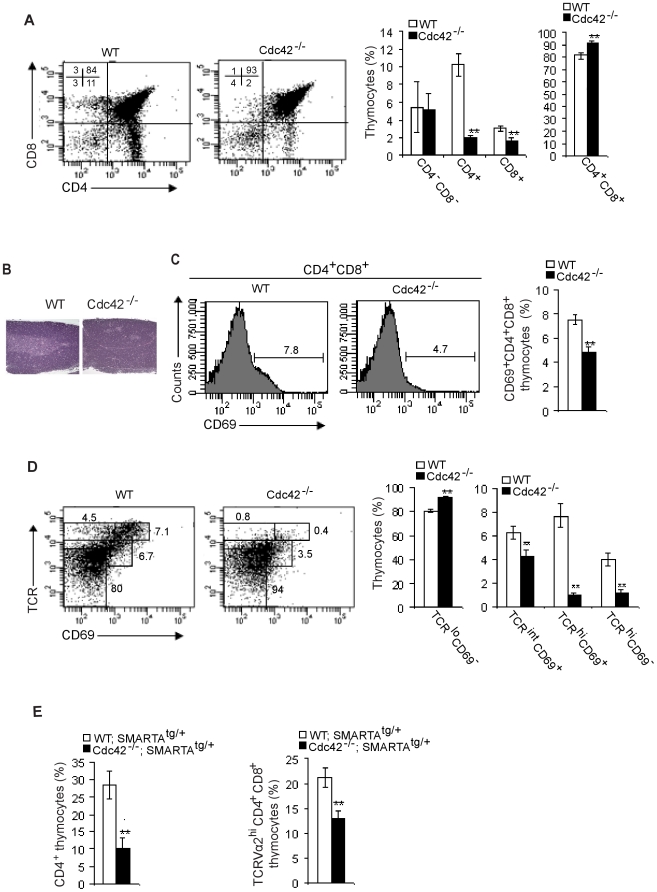
Defective positive selection in Cdc42^−/−^ thymocytes. (A) Flow cytometry of thymocytes from wild type (WT) and Cdc42^−/−^ mice. Numbers in dot plots indicate percent cells in each corresponding quadrant; right, average frequency of thymocyte subsets. n = 12. **P<0.01. Error bars represent SD. (B). Thymus sections from WT and Cdc42^−/−^ mice, stained with hematoxylin and eosin. The medulla exhibits lighter staining. Data are representative of 3 mice. (C) Flow cytometry of the expression of CD69 in WT and Cdc42^−/−^ DP thymocytes. Numbers above bracketed lines indicate percent CD69^+^ cells; right, average frequency of CD69^+^ DP cells. n = 5. **P<0.01. Error bars represent SD. (D) Flow cytometry of CD69 and TCRβ on total thymocytes from WT and Cdc42^−/−^ mice. Numbers adjacent to outlined areas indicate percent cells in each gate; right, average frequency of thymocyte subsets gated at left. n = 5. **P<0.01. Error bars represent SD. (E) Average frequency of CD4^+^ SP thymocytes and TCR Vα2^hi^ DP thymocytes from WT;SMARTA^tg/+^ and Cdc42^−/−^;SMARTA^tg/+^ mice. n = 5. **P<0.01. Error bars represent SD.

To examine the role of Cdc42 in multiple stages of T cell development, we first characterized thymopoiesis in Cdc42^−/−^ mice. By flow cytometry analysis (FACS), we found that the proportion and numbers of DP thymocytes were increased and SP thymocytes were decreased in Cdc42^−/−^ mice (Figure 1A) [11]. Consistent with these changes, the cortex of Cdc42-deficient thymus was prominent while the medulla of mutant thymus was small and inconspicuous (Figure 1B). Because the cortex is a place where DP thymocytes reside and undergo selection, we next examined thymocyte positive selection in Cdc42^−/−^ mice. The percentage of DP thymocytes expressing CD69, a marker upregulated in positively-selected thymocytes, was significantly reduced in Cdc42 null mice, compared to that in wild-type (WT) mice (Figure 1C). To determine at which point thymocyte development is blocked in Cdc42^−/−^ mice, we analyzed expression of TCRβ together with CD69 in total thymocytes, by FACS. As shown in Figure 1D, the percentage of TCR^lo^CD69^−^ cells, which correspond to preselection DP thymocytes, was higher in Cdc42^−/−^ mice. The percentage of TCR^int^CD69^+^, TCR^hi^CD69^+^, and TCR^hi^CD69^−^ cells, which contain mainly DP thymocytes at the initial stage of positive selection, immature SP thymocytes, and mature SP thymocytes, respectively, was lower in Cdc42^−/−^ mice. These results suggest that Cdc42-deficient thymocytes were blocked at the earliest stage of positive selection.

To reaffirm the inhibitory effect of Cdc42 deficiency on thymocyte positive selection, we crossed Cdc42^−/−^ mice with SMARTA TCR transgenic mice. SMARTA TCR transgenic mice express TCRV_α_2Vβ8 that specifically recognizes lymphocytic choriomeningitis virus (LCMV) epitope gp61–80 presented by class II major histocompatibility complex (MHC) molecule I-A^b^ [23]. Thus, the resultant Cdc42^−/−^;SMARTA^tg/+^ from Cdc42^−/−^ crossbreeding with SMARTA mice are dominated with monoclonal CD4^+^ T cells. FACS analysis of thymocytes from Cdc42^−/−^;SMARTA^tg/+^ mice revealed fewer CD4^+^ SP thymocytes and lower expression of Vα2 TCR in DP thymocytes, compared to WT;SMARTA^tg/+^ mice (Figure 1E). Taken together, these results show that Cdc42 is required for thymocyte positive selection and maturation.

### Cdc42 knockout impairs migration, survival, and TCR signaling of thymic T cells

**Figure 2:**
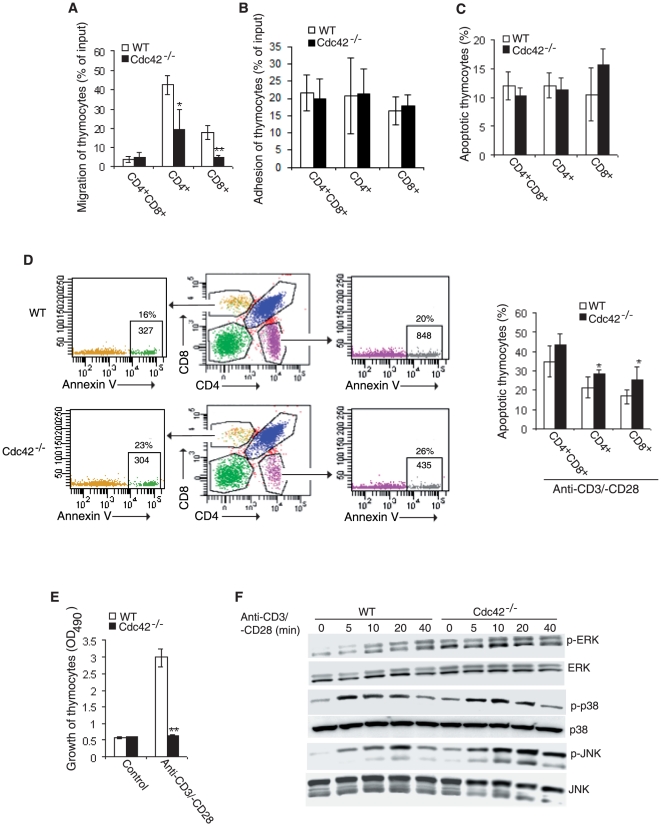
Defective migration, survival, and TCR signaling in Cdc42^−/−^ thymocytes. (A) Migration to SDF-1α of DP thymocytes or to MIP3β of SP thymocytes from wild type (WT) and Cdc42^−/−^ mice. Data were expressed as numbers of cells migrated to the exterior of transwell chambers relative to numbers of cells initially seeded in the interior of transwell chambers (% of input)n = 5. *P <0.05; **P <0.01. Error bars represent SD. (B) Adhesion to fibronectin of thymocytes from WT and Cdc42^−/−^ mice. Data were expressed as numbers of adhered cells relative to numbers of cells plated (% of input). n = 6. Error bars represent SD. (C) Apoptosis of Ex vivo thymocytes from WT and Cdc42^−/−^ mice. Freshly isolated thymocytes were stained with anti-CD4 and -CD8 antibodies followed by Annexin V staining. The cells were then analyzed by flow cytometry. n = 4. *P <0.05. Error bars represent SD. (D) Apoptosis of cultured thymocytes from WT and Cdc42^−/−^ mice. Isolated thymocytes were cultured for 24 hours with anti-CD3/-CD28 antibodies and stained with anti-CD4 and -CD8 antibodies followed by Annexin V staining. The cells were then analyzed by flow cytometry. Numbers outside the gates indicate percent cells in each gate and numbers inside the gates indicate absolute numbers of Annexin V^+^ cells analyzed in each gate; right, average frequency of Annexin V^+^ thymocytes. Data are representative of three independent experiments. n = 4. *P <0.05. Error bars represent SD. (E) Anti-CD3/-CD28-induced proliferation of thymocytes from wild type (WT) and Cdc42^−/−^ mice. TCRβ^+^ thymocytes were plated on 96-well plates at 1×10^6^/well in 200 µL culture media in the presence or absence of plate-coated anti-CD3 (10 µg/mL) plus soluble anti-CD28 (2 µg/mL). The cells were cultured for 3 days and assayed for growth rate. n = 6. **P <0.01. Error bars represent SD. (F) MAP kinase activities in thymocytes from WT and Cdc42^−/−^ mice. TCRβ^+^ thymocytes were stimulated with or without anti-CD3 (10 µg/mL) and -CD28 (2 µg/mL) for indicated time. Western blotting was performed to assess the phosphorylation status of Erk, p38, and JNK. The result is a representative of three experiments.

Cell migration and adhesion are known to be critical for thymopoiesis [1]. We found that depletion of Cdc42 dampened migratory activity of SP thymocytes toward MIP-3β (Figure 2A). However, adhesion of Cdc42-deficient SP thymocytes to fibronectin remained unchanged (Figure 2B). These data suggest that Cdc42 is important for SP thymocyte migration but not adhesion and that defective migration may underlie the developmental block in Cdc42^−/−^ thymocytes.

Because Cdc42 can regulate cell survival [11], we next examined the survival status of Cdc42^−/−^ thymocytes. To our surprise, Annexin V staining of freshly isolated thymocytes did not reveal a survival defect in Cdc42^−/−^ DP and SP thymocytes (Figure 2C). However, we found that disruption of Cdc42 led to a minor but statistically significant increase in apoptosis in SP thymocytes that were stimulated in vitro by CD3 and CD28 antibodies (Figure 2D). Thus, Cdc42 may be involved in protecting thymocytes from anti-CD3-induced apoptosis.

The defect in thymic T cell development in Cdc42^−/−^ mice suggests a perturbation in thymocyte TCR signaling. Indeed, we found that TCR ligation-induced thymocyte growth was abrogated in the absence of Cdc42 (Figure 2E). Moreover, TCR engagement-triggered ERK and JNK MAPK activation was enhanced in Cdc42^−/−^ thymocytes (Figure 2F). However, ablation of Cdc42 had no effect on TCR-induced p38 activation (Figure 2F). These findings are contrary to some of the previous reports of a positive role of Cdc42 in the regulation of ERK, JNK, and/or p38 activities and reflect the cell type-specific signaling function of Cdc42 [24].

### Cdc42 is required for TCR clustering and actin polarization during the formation of mature IS in peripheral naive T cells

**Figure 3:**
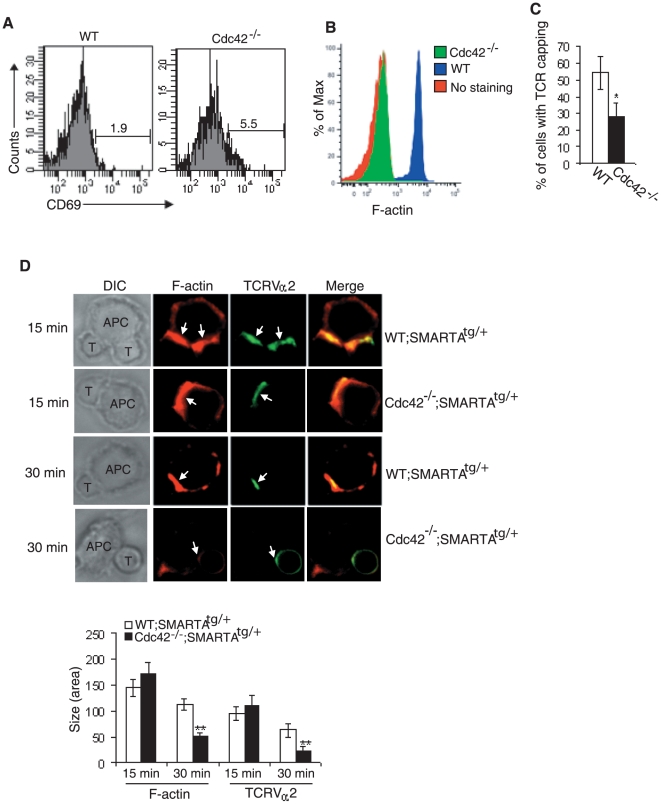
Defective TCR clustering and actin polymerization in Cdc42^−/−^ peripheral naive T cells during mature immunological synapse formation. (A) Expression of T cell activation marker CD69 in naïve CD4^+^ T cells from wild type (WT) and Cdc42^−/−^ mice. Splenocytes were stained with anti-CD4, -CD62L, -CD44, and -CD69 antibodies. CD69 expression in CD4^+^ CD62L^+^CD44^−^ naïve T cells was analyzed by flow cytometry. Data are representative of five mice (B) Anti-TCR-induced actin polymerization in naïve CD4^+^ T cells from WT and Cdc42^−/−^ mice. Naive CD4^+^ T cells from spleen were incubated with anti-TCRβ antibody on ice and stimulated with anti-hamster IgG at 37°C. Cells were fixed, permeabilized, and incubated with FITC-phalloidin, and analyzed by flow cytometry. Data are representative of four mice. (C) Anti-TCR-induced TCR capping/clustering in naïve CD4^+^ T cells from WT and Cdc42^−/−^ mice. Naïve CD4^+^ T cells from spleen were seeded on poly-L-lysine-coated slides, incubated with anti-TCRβ antibody on ice, and stimulated with biotin-conjugated anti-hamster IgG at 37°C. After fixation, cells were stained with Avdin-Texas Red and visualized with a Zeiss fluorescence microscope Average percentage of capped cells was obtained by counting cells from 6 random fields. Data are representative of 3 mice. (D) Antigen-presenting cells (APCs)-induced actin polymerization and TCR clustering in naïve T cells from WT;SMARTA^tg/+^ and Cdc42^−/−^;SMARTA^tg/+^ mice. Splenic naïve CD4^+^ T cells bearing SMARTA transgenic TCRV_α_2Vβ8 were incubated with gp61-80-loaded APCs (CHB.2 B cells) for 15 min or 30 min, fixed, permeabilized, stained with phalloidin (red) and anti-TCRV_α_2 (green), and visualized with a Leica immunofluorescence microscopy. Differential interference contrast (DIC) show APC-T cell conjugates. Arrowheads point to F-actin and TCR clusters at interface between APC and T cells. Data are representative of 20 to 30 APC-T cell conjugates. The size (area) of F-actin and TCR clusters was quantified by Image J.

Mature SP thymocytes emigrate from thymus to peripheral tissues where they are maintained as naïve T cells. Upon binding of TCR with antigen-MHC complex, naïve T cells differentiate to effector and memory cells to exert their immune functions [1], [2]. The differentiation of effector and memory T cells requires naïve T cell activation [1], [2]. Previously we showed that Cdc42 deficiency in naïve T cells resulted in a hyperactive phenotype [11]. In support of this, we found that T cell activation marker CD69 was upregulated in Cdc42^−/−^ naïve T cells (Figure 3A) [11], in contrast to the downregulation of CD69 in Cdc42^−/−^ DP thymocytes. This seemingly paradoxical observation could reflect a distinct function of CD69 in peripheral T cells from that in thymocytes where CD69 is a marker for positive selection. The data suggest that Cdc42 plays a developmental stage-specific role in regulating CD69 expression. Upon further examination of the early events in the course of naïve T cell activation we found that anti-TCR-induced actin polymerization was impaired in Cdc42^−/−^ naïve T cells (Figure 3B), consistent with the established role of Cdc42 in actin cytoskeleton reorganization. TCR cap is an asymmetric membrane structure that is formed through ligand-induced TCR clustering, depending on actin cytoskeletal rearrangement [25]. Anti-TCR stimulated TCR capping/clustering activity was reduced by ∼50% in Cdc42 null naïve T cells (Figure 3C). To determine if Cdc42 deficiency also causes defects in actin polymerization and TCR clustering during immune synapse formation, we mixed Cdc42^−/−^;SMARTA^tg/+^ or WT;SMARTA^tg/+^ naïve T cells with APCs preloaded with LCMV epitope gp61-80, incubated cell mixture for 15 or 30 min, and stained F-actin and TCRVα2. Actin polymerization and TCR clustering at the interface between Cdc42^−/−^;SMARTA^tg/+^ T cells and APCs were impaired at 30 min after mixing T cells with APCs, as reflected by a marked reduction of the size of polarized F-actin and TCR clusters in Cdc42^−/−^;SMARTA^tg/+^ T cells (Figure 3D). Interestingly, Cdc42^−/−^;SMARTA^tg/+^ T cells appeared to undergo normal actin polymerization and TCR clustering at 15 min after mixing T cells and APCs (Figure 3D). Since TCR clusters initially form at the periphery of immature IS and subsequently translocate to the center of IS to facility IS maturation [26], [27], [28], [29], our data suggest that Cdc42 is not required for TCR microcluster formation but essential for its centralization during the maturation of IS.

### Cdc42 deficiency enhances effector and memory T cell differentiation

**Figure 4:**
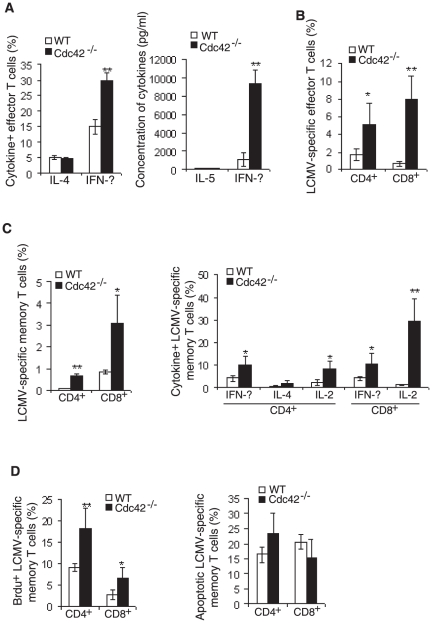
Enhanced effector and memory T cell differentiation in the absence of Cdc42. (A) Th1 and Th2 effector cell differentiation in vitro from wild type (WT) and Cdc42^−/−^ naïve T cells. Splenic CD4^+^ naïve T cells were cultured under Th1 or Th2 polarized conditions. At day 3, levels of Th2-produced IL-5 and Th1-produced IFN-γ in culture supernatants were quantified by ELISA (right). At day 6, the cells were restimulated as described in the Methods. IL-4-secreting Th2 cells and IFN-γ-secreting Th1 cells were analyzed 6 hours after restimulation, by flow cytometry (left). n = 4. **P <0.01. Error bars represent SD. (B) Effector cell differentiation in vivo in WT and Cdc42^−/−^ mice. WT and Cdc42^−/−^ mice were administrated with lymphocytic choriomeningitis virus (LCMV). At day 9, frequency of LCMV-specific CD4^+^ and CD8^+^ effector cells were analyzed by flow cytometry, using respective tetramer staining reagents. n = 6. *P<0.05, **P<0.01. Error bars represent SD. (C) Memory cell differentiation in vivo in WT and Cdc42^−/−^ mice. WT and Cdc42^−/−^ mice were administrated with LCMV. At day 50, frequency of LCMV-specific CD4^+^ and CD8^+^ memory cells were analyzed as described in (B). Frequency of cytokine-secreting LCMV-specific CD4^+^ and CD8^+^ memory cells were analyzed after in vitro restimulation with gp61-80 and gp33-41, respectively. n = 6. *P<0.05, **P<0.01. Error bars represent SD. (D) Memory cell proliferation and survival in WT and Cdc42^−/−^ mice. WT and Cdc42^−/−^ mice were injected with Brdu before being sacrificed at day 50 post LCMV infection. Brdu^+^ LCMV-specific CD4^+^ and CD8^+^ memory cells were analyzed by flow cytometry. Survival status of LCMV-specific CD4^+^ and CD8^+^ memory cells were analyzed by Annexin V staining.

Cdc42 deficiency causes naïve T cell hyperactivation (Figure 3A), raising a possibility that subsequent effector and memory T cell differentiation may be altered in the absence of Cdc42. To examine this, we first cultured CD4^+^ naïve T cells from Cdc42^−/−^ and WT mice in vitro under standard Th1- and Th2-polarizing conditions, and analyzed IFN-γ and IL-4/IL-5 production, respectively. Disruption of Cdc42 led to more IFN-γ-producing cells and IFN-γ secretion (Figure 4A). However, the frequency of IL-4-producing cells and production of IL-5 were not altered in the absence of Cdc42 (Figure 4A).

We next examined effector cell differentiation in vivo. To this end, Cdc42^−/−^ and WT mice were inoculated with LCMV and sacrificed 9 days later and analyzed for LCMV-specific CD4^+^ and CD8^+^ T cells, using MHC class II and class I tetramers containing LCMV epitopes, I-A^b^gp61-80 and D^b^gp33-41, respectively. We found that compared to WT mice, Cdc42^−/−^ mice had higher frequency of LCMV-specific (tetramer^+^) CD4^+^ and CD8^+^ effector cells (Figure 4B). This increase persisted when the cells transited into the memory compartment, as frequency of LCMV-specific CD4^+^ and CD8^+^ T cells remained higher in mutant mice by 50 days after infection (Figure 4C). Moreover, while IL-4-producing CD4^+^ memory cells remained unchanged in the absence of Cdc42, IFN-γ- and IL-2-producing CD4^+^ and CD8^+^ memory cells were increased in Cdc42^−/−^ mice. In vivo Brdu labeling experiment revealed that LCMV-specific Cdc42^−/−^ memory T cells had a significantly increased proliferative activity (Figure 4D). Surprisingly, Cdc42 deficiency did not alter survival of LCMV-specific memory cells (Figure 4D). These results suggest that Cdc42 restrains proliferation and differentiation of CD8^+^ and CD4^+^ effector and memory cells.

### Cdc42 deficiency causes an exacerbated liver damage in mice

**Figure 5:**
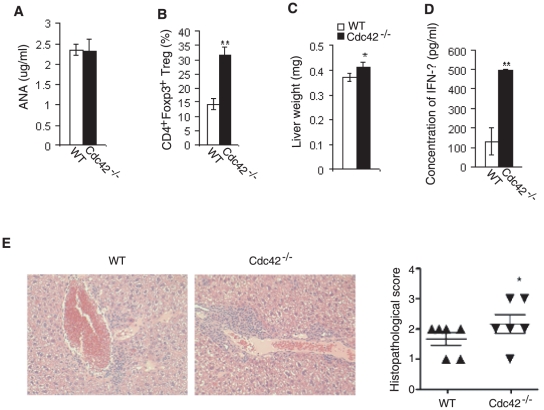
Exacerbated liver damage in Cdc42^−/−^ mice. (A) Level of anti-nuclear autoantibody (ANA) in serum from wild type (WT) and Cdc42^−/−^ mice. Peripheral blood was collected by tail vein bleeding and serum was prepared and analyzed for ANA by ELISA. n = 5. Error bars represent SD. (B) Regulatory T cells in WT and Cdc42^−/−^ mice. Splenocytes were stained for CD4 followed by intracellular staining of Foxp3. CD4^+^Foxp3^+^ regulatory T cells were analyzed by flow cytometry. n = 5. **P<0.01. Error bars represent SD. (C) Weight of livers from WT and Cdc42^−/−^ mice. WT and Cdc42^−/−^ mice were injected with Novosphingobium aromaticivorans (N. aro). Six weeks after N.aro infection, mice were sacrificed and livers were weighed. n = 6. *P<0.05. Error bars represent SD. (D) IFN-γ production from WT and Cdc42^−/−^ splenocytes. WT and Cdc42^−/−^ mice were injected with N. aro. Six weeks post infection, splenocytes were harvested from WT or Cdc42^−/−^ mice and cocultured with bone marrow-derived dentritic cells (DC) prepulsed with N.aro (N. aro to DC: 5∶1). Cell cultures were assayed 72 hours later for the release of IFN-γ by ELISA. n = 6. **P<0.01. Error bars represent SD. (E) Portal inflammation in livers from WT and Cdc42^−/−^ mice. Six weeks post N.aro infection, livers from WT and Cdc42^−/−^ mice were sectioned, stained by hematoxylin and eosin, and evaluated microscopically (left) for leukocytic and lymphocytic infiltration. Liver lesions were scored (right) by examining 5 sections separated by 25 µM for portal inflammation using the following scale: 0 =  no inflammation, 1 =  sparse mononuclear cell infiltrates, 2 =  moderate inflammation, 3 =  intense inflammation, 4 =  intense inflammation and spillover into the periportal parenchyma. The score was based on the most severe infiltration observed in the majority of portal fields. Statistical significance was calculated using a Mann-Whitney test based on exact p-value computations to account for ties. n = 6. *P<0.05. Error bars represent SE.

Aberrant effector and memory T cell differentiation may lead to autoimmune responses. We thus investigated if Cdc42^−/−^ mice showed autoimmune phenotypes. The serum level of anti-nuclear autoantibodies (ANA) in Cdc42^−/−^ mice was comparable to that in WT mice (Figure 5A), suggesting that no spontaneous autoimmunity is developed in mutant mice. This could attribute to an increased frequency of regulatory T cells (Figure 5B).

We next examined the role of Cdc42 in an induced liver-specific inflammatory disease model. Cdc42^−/−^ and WT mice were inoculated with alphaproteobacterium N. aro that induces antibodies against microbial Pyruvate Dehydrogenase Complex E2 (PDC-E2) and its mitochondrial counterpart [30]. The mice were analyzed 6 weeks after infection. We found that livers from Cdc42^−/−^ mice were moderately larger than that from WT mice after N. aro challenge (Figure 5C). Strikingly, splenocytes from N.aro-infected Cdc42^−/−^ mice produced 3-fold more IFN-γ when restimulated in vitro with bone marrow-derived dentritic cells preloaded with N.aro (Figure 5D). Moreover, histological examination of livers from Cdc42^−/−^ mice revealed exacerbated portal inflammation with increased leukocytic and lymphocytic infiltration (Figure 5E). These data suggest that Cdc42 suppresses inflammation in this mouse model.

## Discussion

In this study, we report that Cdc42 deficiency blocks thymopoiesis at DP stage, resulting in a decreased production of SP thymocytes. We further show that disruption of Cdc42 causes a dampened actin polymerization and TCR clustering in naïve T cells during the formation of mature IS. Moreover, ablation of Cdc42 induces enhanced naïve T cell differentiation to effector and memory cells in vitro and in vivo. Lastly, we detected more severe liver damage in Cdc42-deficient mice in a liver-specific autoimmune disease model. Our findings suggest that while Cdc42 is required for thymopoiesis, it plays a restrictive role in effector and memory T cell differentiation and autoimmunity.

Cdc42 regulates thymopoiesis through complex mechanisms. First, Cdc42^−/−^ DP thymocytes expressed less CD69, suggesting that positive selection is impaired in mutant mice. Given that Cdc42^−/−^ mice have more preselection DP thymocytes, T cell development in mutant mice appears to be impeded at early stage of positive selection. Second, Cdc42^−/−^ SP thymocytes are dampened in migration. Thirdly, Cdc42^−/−^ SP thymocytes were more susceptible to anti-CD3-induced cell death. Finally, Cdc42^−/−^ thymocytes display a defective proliferation, in response to anti-CD3/-CD28 stimulation, and aberrant TCR signaling. Therefore, a combined effect of Cdc42 on positive selection, migration, survival, growth, and TCR signaling contributes to its regulation of thymic T cell development. It is noted that contrary to a positive role of Cdc42 in the regulation of ERK, JNK, and/or p38 activities reported in some of the previous studies, ERK and JNK activities were enhanced while p38 activity remained unchanged in response to TCR stimulation in Cdc42^−/−^ thymocytes. Given that ERK is known to promote thymocyte positive selection [31], the elevated ERK activity could be due to a compensatory effect for the compromised positive selection in Cdc42^−/−^ thymocytes. On the other hand, since JNK positively regulates thymocyte negative selection [32], the increased JNK activity could be suggestive of an enhanced negative selection in Cdc42^−/−^ thymocytes. These results, in the context of diverse signaling functions of Cdc42 [24], are consistent with the notion that Cdc42 plays a cell type-specific role in regulating ERK, JNK, and p38 activities. The thymopoietic phenotypes of Cdc42^−/−^ mice are reminiscent of and complementary to the previous observations in mouse models deficient in Rac1, Rac2 or RhoH, other members of Rho family GTPases. In Rac1 and Rac2 double knockout mice, T cell development is severely impaired at checkpoints of β selection and positive selection. The developmental block is associated with defects in thymocyte proliferation, survival, adhesion, and migration. Further, loss of Rac1 and Rac2 suppresses TCR-mediated interleukin-2 production and Akt activation and leads to hyperactivation of Notch signaling [1], [33]. Similarly, RhoH knockout mice display a defective T cell maturation during transition from DN3 to DN4 and during positive selection. And RhoH deficiency leads to defective TCR signaling manifested by decreased activation of CD3ζ, LAT, PLCγ, Vav1, and Erk, and by reduced calcium influx [34], [35].

Cdc42 is well appreciated as a key regulator of actin cytoskeleton rearrangement [36], [37]. Consistent with this view, Cdc42 deficiency diminishes anti-TCR-induced actin polymerization of naïve T cells. Moreover, anti-TCR-induced TCR capping/clustering is attenuated in Cdc42^−/−^ naïve T cells. Essentially, by examining Cdc42^−/−^ transgenic monoclonal T cells, we found that actin polymerization and TCR clustering were impaired at later but not early stage of IS formation with APCs. In line with recent studies showing that TCR microclusters initially emerge at the periphery of IS and then move to the center of IS, resulting in mature IS formation [26], [27], [28], [29], it is logical to reason that Cdc42 may promote centralization but not emergence of TCR microclusters. Nonetheless, the hypothesis warrants further and stringent examination in the future. It is not clear at this point why Cdc42 deficiency has no effect on TCR microcluster formation. However, in view that TCR microcluster formation is dependent on actin polymerization and that Rac1, along with Cdc42, plays a key role in actin polymerization [38], [39], Rac1 may compensate for the loss of Cdc42 to promote TCR microcluster formation during initial phase of IS formation. It was originally proposed that IS promoted TCR signaling transduction [40]. However, most recent studies suggest that IS plays a dual role in T cell activation: TCR microclusters at the periphery of IS initiate TCR signaling transduction, whereas centralized TCR microclusters are degraded, resulting in TCR signaling termination [26], [27], [28], [29]. We thus postulate that while being triggered normally, TCR signaling could not be terminated in Cdc42^−/−^ cells. This hypothesis could potentially explain sustained ERK activation and hyperactive phenotypes observed in Cdc42^−/−^ T cells [11]. The phenotypes of TCR cluster dynamics caused by Cdc42 deficiency is reminiscent of that resulted from inhibition of myosin IIA showing impaired TCR microcluster translocation to the center of IS, with normal TCR microcluster formation [41]. Since we detected an impaired activation of myosin light chain in Cdc42^−/−^ T cells (data not shown), it is possible that Cdc42 functions upstream of myosin IIA in the regulation of TCR microcluster kinetics.

In an in vitro CD4^+^ effector T cell differentiation system, we found that Th1 cell differentiation was enhanced in the absence of Cdc42. However, we found no evidence of differences in Th2 cell differentiation. Considering that Cdc42^−/−^ T cells bear increased level of TCR signaling [11], our data are in support of the literatures that strong TCR signals induce Th1 cell differentiation [3], [42], [43]. The differentiation of Th1 effector cells are mainly governed by JAK1/2/STAT1/3/4 signaling pathway and transcriptional factor T-bet [3], [4]. In this context, further work is needed to determine if Cdc42 deficiency influences intracellular signaling pathways that are discrete for Th1 cells. Of note, the role of Cdc42 in Th cell differentiation are in sharp contrast to that of Cdc42 immediate downstream effector WASP, disruption of which leads to a diminished Th2 response while has no effect on Th1 cells [44]. This suggests that WASP is independent of Cdc42 in Th cell differentiation.

In an in vivo LCMV mouse model, we repeatedly detected an increase in CD4^+^ effector cells in Cdc42^−/−^ mice. CD8^+^ effector cells are also increased in mutant mice. Further, Cdc42^−/−^ mice generate more LCMV-specific memory cells, compared to WT mice. Consistent with the effects of Cdc42 deficiency on Th effector cell differentiation, Cdc42^−/−^ CD4^+^ memory cells produce more Th1 signature cytokines IFN-γ and IL-2, whereas Th2 signature cytokine IL-4 remains comparable in Cdc42^−/−^ and WT memory cells. The increased memory cells in Cdc42^−/−^ mice are attributable to enhanced proliferative renewal. In contrast to an increased apoptosis in Cdc42^−/−^ naïve T cells [11], there is no survival defect in Cdc42^−/−^ memory cells. This is an interesting observation as it assigns a developmental stage specific role of Cdc42 in T cell survival regulation.

Cdc42 deficiency results in a constitutive activation of naïve T cells, which may predispose Cdc42^−/−^ mice to autoimmune diseases [45]. However, serum anti-nuclear autoantibodies are maintained at basal level in Cdc42^−/−^ mice. This self-tolerance might be associated with increased regulatory T cells. It would be interesting to assess how regulatory T cells act on memory phenotype T cells to suppress the development of spontaneous autoimmunity in Cdc42^−/−^ mice. One potential mechanism could be that regulatory T cells kill memory phenotype T cells by producing granzyme and/or perforin [46]. Albeit Cdc42^−/−^ mice do not appear to develop spontaneous autoimmunity in the absence of infectious agents, they indeed mount more severe autoimmune responses in a N.aro-induced and T cell-mediated liver-specific autoimmune disease model. The mice have increased incidence of liver portal inflammation and splenocytes from the mice produce higher level of IFN-γ upon restimulation with N. aro. These phenotypes could result from pre-activated states of Cdc42^−/−^ T cells that hold lower threshold for antigen-induced T cell activation.

In conclusion, we demonstrate that Cdc42 positively regulates thymocyte development but negatively regulates naïve T cell activation and differentiation to effector and memory T cells. Thereby, Cdc42 plays a stage-specific role in T cell development. Our data provide a novel insight into the mechanisms of T cell development and adaptive immunity.

## Materials and Methods

### Ethics statement

This study involved using mice. The study was carried out in strict accordance with the recommendations in the Guide for the Care and Use of Laboratory Animals of the Cincinnati Children's Hospital Research Foundation. The protocol was approved by the Committee on the Ethics of Animal Experiments of the Cincinnati Children's Hospital Research Foundation (permit Number: 8D06052). Mice were anesthetized when necessary, using ketamine (80–100 mg/kg im), aceptromazine (4–6 mg/kg im) and atropine (0.1 mg/kg im). Anesthesia was maintained using ketamine (30 mg/kg im) as needed. During the course of experiments, mice were isolated in microisolator cages and cared for in the Laboratory Animal Resource Center by trained technician and two veterinarians. Animals were checked daily by qualified personnel in the lab. The method of euthanasia used was CO_2_ euthanasia. This method was approved by the Animal Care and Use Committee of the Cincinnati Children's Hospital Research Foundation and consistent with the recommendations of the Panel on Euthanasia of the American Veterinary medical Association.

### Generation of mice lacking Cdc42 in T lymphocytes

Conditional targeted Cdc42 *^flox/flox^* mice were generated as described previously [17]. The flox allele contains loxP sites flanking exon 2 of Cdc42 alleles. To delete Cdc42 in vivo in T cell lineage, Cdc42 *^flox/flox^* mice were mated with mice expressing Cre recombinase under the control of Lck proximal promoter (from Jackson Laboratory). Cdc42 *^flox/flox^*;Lck-Cre mice were crossed with SMARTA mice expressing transgenic TCR (Vαβ2Vβ8) to generate Cdc42^−/−^;SMARTA^tg/+^ compound mice [23]. All mice were housed under specific pathogen-free conditions in the Animal Facility at Cincinnati Children's Hospital Research Foundation. All mice used were 4–8 weeks of age.

### Flow cytometry analysis

Single cell suspensions were prepared from thymus. Cells were incubated for 20 min at room temperature with various combinations of the following cell-surface marker antibodies: anti-CD4, anti-CD8, anti-CD69, anti-TCRβ (H57-597), and anti-TCR Vα2 (BD Biosciences). Immunolabeled cells were analyzed by flow cytometry on a FACSCanto system using FACSDiVa software (BD Biosciences).

### Histopathology

Thymuses and livers were fixed in 10% buffered formalin, embedded in paraffin, sectioned (5 µM), and stained with hematoxylin and eosin.

### Cell migration assay

Cell migration was measured using a transwell chamber. Dulbecco modified Eagle medium (DMEM) containing 500 ng/mL SDF-1 (PEPROTECH, Rocky Hill, NJ) or MIP-3β (R&D System, Minneapolis, MN) was added to the exterior of the transwell chamber. Flow cytometry sorted CD4^+^CD8^+^ DP, CD4^+^ or CD8^+^ SP thymocytes were suspended in the DMEM and added to the interior of the transwell chamber and incubated for 4 hours. Cells migrated through polyester membrane were counted.

### Cell adhesion assay

Flow cytometry sorted CD4^+^CD8^+^ DP, CD4^+^ or CD8^+^ SP thymocytes were plated onto 10 µg/mL fibronectin-coated 96-well plates and incubated for 2 hours. The unattached cells were washed away with Dulbecco modified phosphate-buffered saline. The attached cells were trypsinized and counted.

### Cell apoptosis analysis

Freshly isolated thymocytes or thymocytes treated for 24 hours with anti-CD3/-CD28 antibodies (BD Biosciences) were incubated with anti-CD4 and anti-CD8 antibodies for 20 min. Cells were washed, incubated with Annexin V (BD Biosciences) for 20 min and then analyzed by flow cytometry.

### In vitro Proliferation assay

TCRβ^+^ thymocytes were sorted by flow cytometry, plated on 96-well plates with or without anti-CD3/-CD28 antibodies, and cultured for 3 days. Cell growth rates were assayed by a nonradioactive cell proliferation assay kit (Promega).

### Immunobot

Whole-cell lysates were prepared and separated by 10% SDS-polyacrylamide gel electrophoresis. The expression or activation (phosphorylation) of ERK, JNK, and p38 was probed by using corresponding antibodies (Cell Signaling Technology).

### Actin polymerization assay

Naïve CD4^+^ T cells from spleen were purified by FACS cell sorting. The cells were incubated with anti-TCRβ antibody 1 µg/ml on ice for 30 min. After washing, cells were stimulated with anti-hamster IgG 3 µg/ml at 37°C for 30 min. Cells were fixed with 4% paraformaldehyde, and permeabilized with 0.1% Triton X-100. Cells were then incubated with fluorescein isothiocyanate (FITC)-phalloidin (Sigma) for 60 min, washed, and analyzed by flow cytometry.

### TCR capping

Purified naïve CD4^+^ T cells were seeded on poly-L-lysine-coated 2 chamber slides and incubated with anti-TCRβ antibody 3 µg/ml on ice for 30 min. Cells were washed and then incubated with biotin-conjugated anti-hamster IgG 3 µg/ml at 37°C for 30 min. After fixation, cells were stained with Avidin-Texas Red (BD PharMingen) for 30 min and visualized with a Zeiss fluorescence microscope and percentage of capped cells was quantified.

### T cell-APC conjugation and immunofluorescence staining

CHB.2 B cells, a B cell lymphoma line used as APCs, were preloaded for 12 h with 10 µg/ml of gp61-80 peptide. The cells were then mixed, by brief spinning, with wild type (WT) or Cdc42^−/−^ splenic naïve CD4^+^ T cells expressing SMARTA transgenic TCRV_α_2Vβ8. The cell mixtures were incubated for 15 min or 30 min, plated on poly-L-lysine-coated coverslips, fixed, permeabilized with cytofix and cytoperm buffer (BD Biosciences), and stained for F-actin with Rhodamine phalloidin (Sigma) and TCRV_α_2 with FITC-anti-TCRV_α_2 antibody. Stained cells were imaged with a fluorescence microscope equipped with a 40 x objective lens and a deconvolution system (Leica) ‘driven’ by Openlab software (Improvision) [47]. The size (area) of F-actin and TCRV_α_2 at the interface of 20 to 30 T cell-CHB.2 B cell couples was quantified by using Image J (NIH).

### In Vitro Th1 and Th2 differentiation

Naïve CD4^+^ T cells were sorted from spleen and cultured with plate-bound anti-TCRβ (30 µg/mL) and soluble anti-CD28 (1 µg/mL) antibodies under Th1- or Th2-polarized condition: Th1: recombinant murine (rm) IL-12 (10 ng/mL), and anti-IL-4 antibody (10 µg/mL); Th2: rhIL-2, rmIL-4 (10 ng/mL), and anti-IFN-γ antibody (10 µg/mL). Cytokines secreted to culture supernatant were collected on day 3 and analyzed using standard enzyme-linked immunosorbent assay (ELISA). The cells were restimulated for 6 hours on day 6 with plate-bound anti-TCRβ antibody (10 µg/mL) in the presence of monesin (Sigma). The cells were then subjected to intracellular cytokine staining following fixation and permeabilization with cytofix and cytoperm buffer [48].

### Detection of antigen-specific T cells

Mice were injected i.p. with 0.25 mL of 2×10^5^ pfu LCMV and administrated with Brdu (500 µg/mouse) once a day for 3 days before sacrifice. Spleens were harvested and single cell suspension was prepared 9 days or 50 days after LCMV injection. LCMV-specific T cells were detected as described previously. Briefly, LCMV-specific CD4^+^ T cells were detected by staining 2×10^6^ splenocytes with I-A^b^gp61-80 tetrameric staining reagents for 2 h at 37°C. During the last 45 min of incubation, cells were stained with antibodies against CD4, CD44, and CD16/32 followed by Annexin V staining or by fixation, permeabilization, and Brdu staining (BD Biosciences). Cells were analyzed for CD4^+^CD44^+^CD16/32^−^tetramer^+^ cells and their survival status and Brdu incorporation, by flow cytometry. LCMV-specific CD8^+^ T cells were detected by staining 2 x 10^6^ splenocytes with D^b^gp33-41 tetrameric staining reagents for 90 min at 4°C. During the last 45 min of incubation, cells were stained with antibodies against CD8 and CD44 followed by Annexin V staining or by fixation, permeabilization, and Brdu staining. Cells were analyzed for CD8^+^CD44^+^tetramer^+^ populations and their survival status and Brdu incorporation, by flow cytometry. Splenocytes collected 50 days after LCMV infection were also cultured with peptides gp61-80 or gp33-41 for 1 hour. Brefeldin A (Sigma) was added to the culture and the cells were further incubated for 3 hours followed by intracellular cytokine staining of CD4^+^ and CD8^+^ cells [49].

### Measurement of anti-nuclear autoantibodies

Blood was collected and serum was prepared and assayed for anti-nuclear autoantibodies (ANA) using an ELISA kit (Alpha Diagnostic).

### Induction of liver-specific autoimmune disease

Bacterial N. aro (5×10^7^) (ATCC) was injected intravenously into 4–7 week old mice on day 0 and day 14. Mice were sacrificed 6 weeks later and analyzed for spleen and liver weight and for liver portal inflammation by hematoxylin and eosin (HE) staining of paraffin-embedded liver sections. Portal inflammation was evaluated microscopically for leukocytic and lymphocytic infiltration. Splenocytes (10^5^) from N.aro-infected mice were also co-cultured with bone marrow-derived dentritic cells (10^5^) prepulsed with N.aro, for 3 days. Cell culture supernatant was collected and assayed for cytokine release by ELISA (R&D Systems, Minneapolis, MN) [30].
